# Population genetics and migration pathways of the Mediterranean fruit fly *Ceratitis capitata* inferred with coalescent methods

**DOI:** 10.7717/peerj.5340

**Published:** 2018-08-07

**Authors:** Maria Belen Arias, Samia Elfekih, Alfried P. Vogler

**Affiliations:** 1 Department of Life Sciences, Silwood Park Campus, Imperial College London, Ascot, United Kingdom; 2 Department of Life Sciences, Natural History Museum, London, United Kingdom; 3 CSIRO Health & Biosecurity, Black Mountain, Canberra, Australia

**Keywords:** Medfly, Migration, Population genetics, Bayesian method, Macrogeographic patterns

## Abstract

**Background:**

Invasive species are a growing threat to food biosecurity and cause significant economic losses in agricultural systems. Despite their damaging effect, they are attractive models for the study of evolution and adaptation in newly colonised environments. The Mediterranean fruit fly, *Ceratitis capitata*, as a member of the family Tephritidae, is one of the most studied invasive species feeding on many fruit crops in the tropics and subtropics worldwide. This study aims to determine the global macrogeographic population structure of *Ceratitis capitata* and reconstruct its potential migration routes.

**Method:**

A partial mitochondrial cytochrome oxidase I gene from >400 individual medflies and 14 populations from four continents was sequenced and subjected to Bayesian demographic modelling.

**Results:**

The Afrotropical populations (Kenya, South Africa and Ghana) harbour the majority of haplotypes detected, which also are highly divergent, in accordance with the presumed ancestral range of medflies in Sub-Saharan Africa. All other populations in the presumed non-native areas were dominated by a single haplotype also present in South Africa, in addition to a few, closely related haplotypes unique to a single local population or regional set, but missing from Africa. Bayesian coalescence methods revealed recent migration pathways from Africa to all continents, in addition to limited bidirectional migration among many local and intercontinental routes.

**Conclusion:**

The detailed investigation of the recent migration history highlights the interconnectedness of affected crop production regions worldwide and pinpoints the routes and potential source areas requiring more specific quarantine measures.

## Introduction

Globalisation and international economic trade have increased the transportation of species outside their natural ranges. Thus, human activities assist the spread of exotic species and increase the rates at which pest species invade new areas (Blackburn et al., 2011; Karsten et al., 2013). The arrival of invasive species is frequently associated with biodiversity losses, changes in ecosystem function, and negative impacts on economy, agriculture and human health (Mack et al., 2000). While they have many adverse effects, invasive species also offer unique opportunities to study evolution and adaptation within entirely different environments compared to their ancestral habitats (Diamantidis, Carey & Papadopoulos, 2008; Vogel et al., 2010).

The Mediterranean fruit fly, *Ceratitis capitata,* also known as medfly, poses a severe economic threat to agriculture, especially fruit production, due to its broad range of more than 260 different host plant species and worldwide distribution (Malacrida et al., 2007). Chronological records and global studies based on genetic markers assume that *C. capitata* populations are subdivided into three different groups: an ancestral population in Sub-Saharan Africa, a younger population in the Mediterranean basin, and various recently derived populations in tropical and subtropical America, Australia and Oceania (Gasperi et al., 1991; Malacrida et al., 2007; Malacrida et al., 1992; Szyniszewska & Tatem, 2014).

Current medfly management approaches vary between countries, although insecticides (baits and full cover-sprays) are the predominant methods used. However, due to harmful effects of insecticides, the Sterile Insect Technique (SIT) using the release of males subjected to sublethal X-ray irradiation is becoming increasingly common (Dyck, Hendrichs & Robinson, 2005). The successful implementation of pest control strategies using SIT relies on information about possible movements and effective population sizes in the regions under management.

Population genetic studies can be used to understand medfly invasion biology by focusing on the degree of subdivision within and among local regions. Additionally, information on demography and dispersal can be inferred from genetic data. However, the interpretation of these data in the medfly is challenging as repeated range expansions and invasions, as well as several cases of regional eradication, have impacted its distribution and genetic diversity. The medfly population genetic structure and invasion routes have been previously studied using various molecular approaches at local (Bonizzoni et al., 2004; Bonizzoni et al., 2001; Elfekih, Makni & Haymer, 2010; Karsten et al., 2013) as well as global level (Bonizzoni et al., 2000; Gasperi et al., 2002; Malacrida et al., 2007). Most of the proposed colonisation routes have been calculated based on traditional methods such as genetic distance or the private-allele method of Slatkin’s (Bonizzoni et al., 2000; Gasperi et al., 2002; Malacrida et al., 1998). Despite these past efforts, the implementation of coalescence methods to investigate medfly invasion have been limited to one study revealing the origin of medfly in Australia (Bonizzoni et al., 2004), and to a another study by Karsten et al. (2015), which used the Approximate Bayesian Computation (ABC) method to show a decrease in genetic diversity outside of Africa, the presumed origin of the introduced range described above. Even though this study provides invaluable genetic information for the medfly colonisation, it provides detailed information on African populations only, therefore, the incorporation of new populations especially in the Palearctic and Neotropical regions is required to improve the current knowledge of medfly dispersal.

In this study, a large-scale phylogeographic analysis was conducted using the cytochrome oxidase gene I (COI) for a pathway analysis of medfly populations across their distribution range. We aim to determine the current macrogeographic population structure of *C. capitata* collected from different populations around the globe, and to reconstruct plausible migration routes using Bayesian coalescence approaches.

## Materials and Methods

### Sample collection

Specimens of *C. capitata* were collected from 11 sites across all biogeographic regions where the species occurs (Afrotropical, Palaearctic, Australasian and Neotropical) (Table 1) between 2009 and 2014. Whole specimens were collected via traps in orchards and reared from infested fruit, and all flies were preserved in 80% ethanol at −20°C until tissue was used for DNA extraction.

Sequences representing populations at further sites (Kenya, Ghana, and Iran) were obtained from GenBank, which was used to increase the sample size at the main collection sites (see Table S1 for details). Below, specimens from the same sampling site will be referred to as a population.

**Table 1:** Collection and sample size for *Ceratitis capitata* included in this study. Details of sample sites location, name of host plant where the individuals were collected and number of individuals collected at each sample site.

**Biogeographic Region**	**Country**	**Location**	**Host**	**Sample size**
Afrotropical	South Africa	Stellenbosch	Guava	37
Palearctic	Egypt	–	–	25
	Israel	Gedera	Fig	19
		Lachish	Orange	19
		Ness Ziona	Orange	19
		Neta’im	Guava	19
		Shefayim	Orange	18
		Yad Mordechai	Lemon	15
	Tunisia	Bizerte	Orange	1
	Greece	Thessaloniki	Apple	20
		Aetolia-Acarnania	Orange	3
	Spain	Valencia	Fig	21
		Malaga	Peach	15
		Murcia	Orange	5
Australasian	Australia	Perth	Orange	24
Neotropical	Guatemala	Santa Barbara	Coffee	51
	Colombia	Cundinamarca	Peach	18
		Nariño	Coffee	4
	Brazil	Salvador	Guava	11
	Peru	Ica	Orange	8

### DNA extraction, sequencing, and alignment

After morphological identification of the collected specimens, genomic DNA was extracted from each specimen using DNeasy Blood & Tissue Spin Column Kit (Qiagen, Valencia, CA, USA). A fragment of the mitochondrial gene cytochrome *c* oxidase subunit I (COI) was amplified using the primers LCO1490 (5′-GGTCAACAAATCATAAAGATATTGG-3′) and HCO2198 (5′-TAAACTTCAGGGTGACCAAAAAATCA-3′) (Folmer et al., 1994). PCRs were conducted in a 20 µl reaction volume, with 0.5 µl of genomic DNA, 0.1 mM dNTPS, 0.5 U/µM BIOTAQ DNA Polymerase, 3 mM MgCl2, 0.3 µM of forward and reverse primers. The PCR program included an initial denaturation step of 94°C for 5 min followed by 35 cycles of 94°C, 30 s; annealing at 51°C for 54 s, 72°C for 54 s and the final extension at 72°C for 7 min. PCR products were sequenced bidirectionally using ABI technology. Sequences were aligned in Geneious software v.7.1.7 (Kearse et al., 2012) together with sequences retrieved from Genbank.

### Genetic diversity and population structure

Levels of genetic diversity were determined estimating the following parameters in DNAsp v.5 and ARLEQUIN v.3.5.2.1 (Excoffier & Lischer, 2010; Librado & Rozas, 2009): the number of haplotypes (*k*), number of segregating sites (S), haplotype diversity (*h*) and nucleotide diversity (*π*). The median-joining (MJ) network (Bandelt, Forster & Röhl, 1999) was used to estimate the genealogical relationships in *C. capitata* haplotypes computed in POPART v.1.7 (Leigh & Bryant, 2015). Population genetic structure was estimated by population pairwise *F*_*st*_. The significant test statistic was performed using 1,000 permutations, and it was computed in ARLEQUIN v.3.5.2.1.

### Demographic inferences

The Tajima’s *D* (Tajima, 1989) and Fu’s Fs (Fu, 1997) statistic tests were performed to identify deviations from neutral models in ARLEQUIN v.3.5.2.1. The past population dynamic through time for the various *C. capitata* haplogroups was inferred using a Bayesian skyline plot method (BSP). Two independent simulations were run using the Hasegawa-Kishino-Yano (HKY) substitution model and uncorrelated lognormal relaxed molecular clock. Each independent run was performed for 5 ×10^7^ Markov chain Monte Carlo (MCMC) iterations (sampled every 1000 iterations) and discarding 10% of the trees as burn-in implemented in BEAST v.2.4 (Drummond & Rambaut, 2007).

In addition to the considerable variation in mutation and substitution rates between genes and taxa, there is also a substantial disparity between mutation rates estimated directly from population studies and those inferred by phylogenetic (species level) studies (Ho et al., 2005). To avoid potential bias defined by the transition between short-term mutation and long-term substitution rate, we compared two molecular rates. The standard invertebrate mitochondrial divergence rate µ =1.15 × 10^−8^ per year (Brower, 1994; Papadopoulou, Anastasiou & Vogler, 2010), and the mutation rate based on *Drosophila melanogaster* laboratory strain estimations µ= 6.2 ×10^−8^ per generation (Haag-Liautard et al., 2008). The latter was used to extrapolate a molecular rate of 4.29 × 10^−7^ for *C. capitata* which has an average of 6.92 generations per year (Diamantidis et al., 2011). Each run was validated in TRACER ensuring a minimum of 200 effective samplings for each statistic. The two-run results were combined using LogCombiner v. 2.4.5 (Drummond & Rambaut, 2007). Finally, the results were visualised by median of skyline plots using TRACER 1.6.

### Migration rate estimates

Connectivity was explored with the software LAMARC v.2.1.10 which estimates demographic parameters such as theta (*θ*), population growth (g) and migration rates (*M*) (Kuhner, 2006). Theta values were estimated as *θ* = 2*μN*_*e*_, where *N*_*e*_ is the effective population size, and µ represents the mutation rate per nucleotide and generation (see below for details). Migration rate was estimated as *M* = *m*∕µ, where *m* is the probability of immigrants per generation and µis the mutation rate per site per generation. The migration rate was multiplied by the *θ* value of the corresponding recipient population to obtain the migrants per generation value (Nm) (Kuhner, 2006). The search strategy consisted of five initial and four final chains; the Bayesian estimation was conducted with ten initial chains with an interval of 20 using a burn-in of 1,000 samples per chain. The analysis results were checked for convergence and effective sample size values (ESS ≥ 200) in TRACER.

## Results

### Genetic diversity and population structure in *Ceratitis capitata*

A total of 403 sequences of *C. capitata* collected in 14 sites distributed worldwide were included in the analysis. The final truncated alignment was 538 bp in length corresponding to 179 amino acids of the mitochondrial COI gene. The number of segregating sites (*S*) within populations ranged from 23 in Kenya and one in Greece (Table 2), and the number of haplotypes (*k*) varied between 18 in Kenya and one and two in Tunisia and Greece, respectively. Tunisia was not included in the further analysis of intra-population diversity because of the lack of haplotype variation. Haplotype diversity (*h*) and nucleotide diversity (*π*) were much higher in the populations from Kenya and South Africa than anywhere else (Table 2), while the number of unique haplotypes was also far higher, despite the overall large amount of specimens analysed in several of the local populations, such as Spain, Guatemala or Israel (Table 2).

**Table 2:** Population genetic diversity indices and neutrality test statistics for *C. capitata*. The indices are shown as *n*: number of samples; *k*: number of haplotypes; *S*: number of segregating sites; *h*: haplotype diversity (with standard deviation SD); *π*: nucleotide diversity (with standard deviation SD). Tajima’s *D* and Fu’s Fs tests were considered statistically significant when ^∗^*P*-value¡0.05, ^∗∗^*P*-value < 0.01 and ^∗∗∗^*P*-value < 0.001.

**Biogeographic region**	**Population**	**Code**	***n***	***k***	**S**	***h*± SD**	**π± SD**	**Tajima’s*D***	**Fu’s Fs**
Afrotropical	Kenya	KE	22	18	23	0.969 ± 0.027	0.0057 ± 0.0034	−1.94**	−15.89***
South Africa	SA	37	17	16	0.941 ± 0.020	0.0053 ± 0.0031	−0.85	−8.56***
	Ghana	GH	5	4	4	0.900 ± 0.161	0.0033 ± 0.0009	−0.41	−1.19
Palearctic	Egypt	EG	25	3	3	0.353 ± 0.112	0.0016 ± 0.0005	0.28	1.15
	Israel	IS	109	3	4	0.072 ± 0.034	0.0002 ± 0.0001	−1.58**	−1.62
	Tunisia	TU	1	1	–	–	–	–	–
	Iran	IR	12	3	2	0.318 ± 0.164	0.0006 ± 0.0003	−1.14	−1.18*
	Greece	GR	29	2	1	0.069 ± 0.063	0.0001 ± 0.0001	−1.02	−2.38
	Spain	SP	42	6	5	0.592 ± 0.068	0.0012 ± 0.0012	−1.69*	−3.34***
Australasian	Australia	AU	24	5	4	0.377 ± 0.122	0.0007 ± 0.0002	0.03	−3.63*
Neotropical	Guatemala	GU	55	10	6	0.766 ± 0.039	0.0024 ± 0.0003	0.25	0.24
	Colombia	CO	22	3	2	0.567 ± 0.051	0.0011 ± 0.0001	0.15	−0.01
	Brazil	BR	12	3	2	0.621 ± 0.087	0.0013 ± 0.0002	0.06	−0.22
	Peru	PE	8	3	2	0.679 ± 0.122	0.0014 ± 0.0003	0.06	−0.22

**Figure 1:**
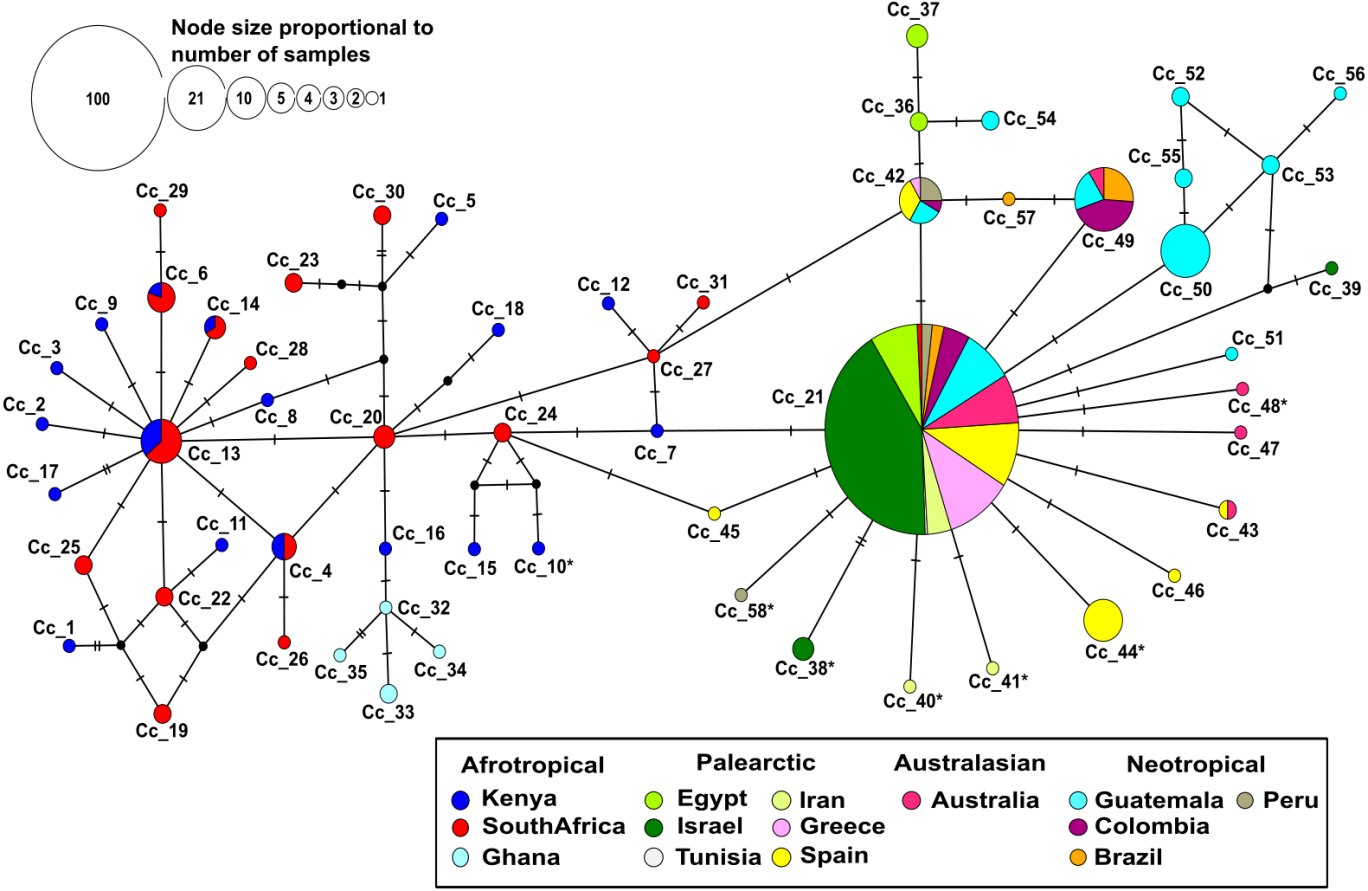
Median-joining network based on 403 individuals of the Mediterranean fruit fly generated using 538 bp of mtDNA COI gene, showing location and frequency of haplotypes. Each circle represents an observed haplotype; the colours reflect sampling location and small black circles indicate unsampled haplotypes inferred from the data. The reticulated network segregated haplotypes according to the different biogeographic region. The more common haplotype in the Afrotropical region cluster is Cc_13 from where singletons are extending outwards. On the other side, the most common haplotype Cc_21 occupies a central position with starburst shape radiation from which the other haplotypes related to Palearctic, Australasian and Neotropical regions are derived. Cc, correspond to *C. capitata.* The * in the haplotype label refers to non-synonymous mutation.

No insertion/deletion or stop codons were detected in the whole data set. Most nucleotide substitutions were synonymous, but six non-synonymous mutations were identified. They corresponded to changes from Methionine to Leucine (Iran), Alanine to Threonine (Peru), Isoleucine to Threonine (Kenya), Proline to Serine (Spain), Valine to Isoleucine (Israel and Australia). All of these changes were mapped to the tips of the haplotype network (Fig. 1).

The median-joining haplotype network contained a total of 58 distinct haplotypes with a low number of ‘unsampled’ (i.e., more than one mutational step apart) haplotypes (Fig. 1). The network was divided into a reticulated portion mainly consisting of Afrotropical haplotypes and a peripheral star-like portion composed of haplotypes from all other locations (Fig. 1). Overall, the Afrotropical haplotypes were more diverse (35 haplotypes from 64 sequences) than those of other regions combined (23 haplotypes from 339 sequences). The Afrotropical cluster was connected to all others via haplotype Cc_21, which was the most frequent haplotype across the dataset (62.28%) and present in almost all localities. This haplotype occupied a central position in the network from which other haplotypes with local distribution in the Palearctic, Australasian and Neotropical regions were derived. Two specimens from South Africa exhibited haplotype Cc_21 and thus constituted a direct link of Afrotropical and other populations.

**Figure 2:**
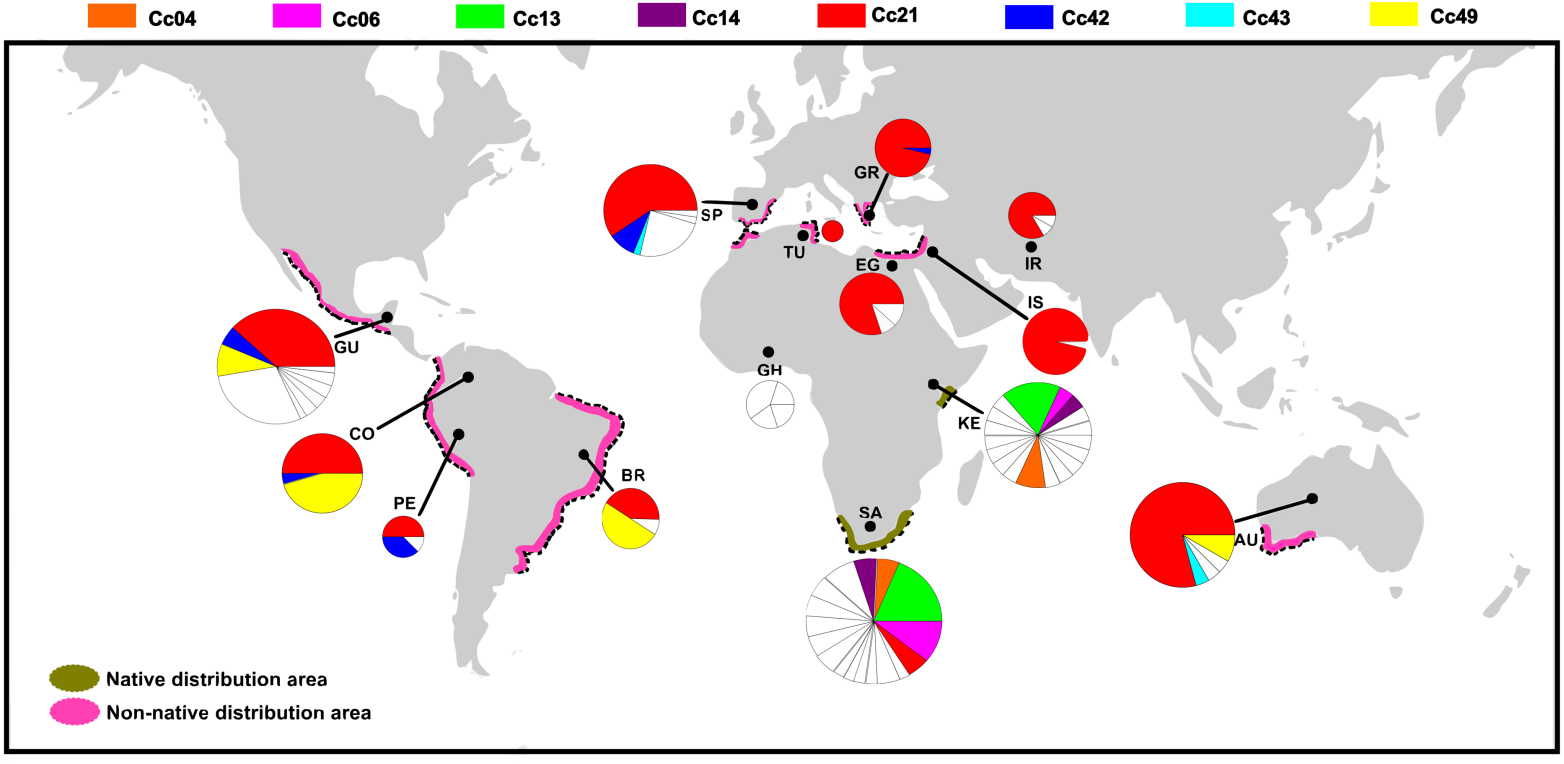
Distribution of COI haplotypes across the study area for *Ceratitis capitata*. The map shows the study locations (country names are abbreviated as in Table 2), and the pie charts indicate the haplotype composition of the population from that location. Each colour represents a shared haplotype found across the study area, and the unique haplotypes (refer to haplotypes found in the samples from one particular population and are absent in the samples from other populations) are uniformly represented in white within pie charts. Native and non-native areas are represented according to Malacrida et al. (2007).

Eight haplotypes were shared between at least two localities (Fig. 2, see the identification code in Table 2), of which the haplotypes Cc_42 and Cc_49 were the most dominant, besides the ubiquitous Cc_21. In the Afrotropical cluster, only the haplotypes Cc_04, Cc_06, Cc_13, and Cc_14 were shared between South Africa and Kenya, but none of them was the centre of an expanded genealogy as the common haplotype Cc_21 in the rest of the world. The pairwise *F*_st_ analysis performed on the 13 localities (Tunisia excluded) showed that the majority of the populations were significantly differentiated (Table 3). Some exceptions were found for neighbouring sites including South Africa and Kenya in the Afrotropical group; Iran, Egypt, Israel and Greece in the Palearctic; or Brazil and Colombia in the Neotropical region. However, some remote sites also presented non-significant differences such as Iran and Australia (Table 3).

**Table 3:** Pairwise *Fst* values between 13 populations of *C. capitata*. The *Fst* values for pairwise comparisons among populations calculated from mtDNA data. Significant tests were performed using 1,000 permutations. Bold values are statistically significant.

** **	**KE**	**SA**	**GH**	**EG**	**IS**	**IR**	**GR**	**SP**	**AU**	**GU**	**CO**	**BR**	**PE**
**KE**													
**SA**	−0.002												
**GH**	**0.058**	**0.074**											
**EG**	**0.346**	**0.302**	**0.492**										
**IS**	**0.694**	**0.608**	**0.854**	**0.146**									
**IR**	**0.315**	**0.278**	**0.474**	−0.009	0.107								
**GR**	**0.516**	**0.439**	**0.772**	**0.079**	−0.007	0.054							
**SP**	**0.236**	**0.210**	**0.312**	**0.088**	**0.313**	0.070	**0.189**						
**AU**	**0.332**	**0.289**	**0.471**	0.004	**0.125**	-0.024	0.062	**0.074**					
**GU**	**0.140**	**0.131**	**0.187**	**0.169**	**0.416**	**0.153**	**0.280**	**0.112**	**0.149**				
**CO**	**0.231**	**0.211**	**0.320**	**0.236**	**0.576**	**0.220**	**0.415**	**0.168**	**0.168**	**0.118**			
**BR**	**0.188**	**0.177**	**0.269**	**0.297**	**0.689**	**0.280**	**0.542**	**0.196**	**0.228**	**0.115**	−0.051		
**PE**	**0.154**	**0.142**	**0.224**	**0.187**	**0.621**	0.168	**0.454**	0.055	**0.166**	0.075	0.161	0.181	

**Notes.**

Population code KE Kenya SA South Africa GH Ghana EG Egypt IS Israel IR Iran GR Greece SP Spain AU Australia GU Guatemala CO Colombia BR Brazil PE Peru

### Demographic history

Across the entire dataset, only six of thirteen sites (Tunisia excluded) were significant for Tajima’s *D* and Fu’s F*s* (Table 2). From the Afrotropical cluster, South Africa and Kenya were highly significant and negative for these neutrality tests. Negative values were also found in the Palearctic (Israel, Iran, Spain) and Australasian regions. These findings may indicate either purifying selection acting on protein coding regions or may be due to recent population expansion that favour a non-random variation of haplotypes.

**Figure 3:**
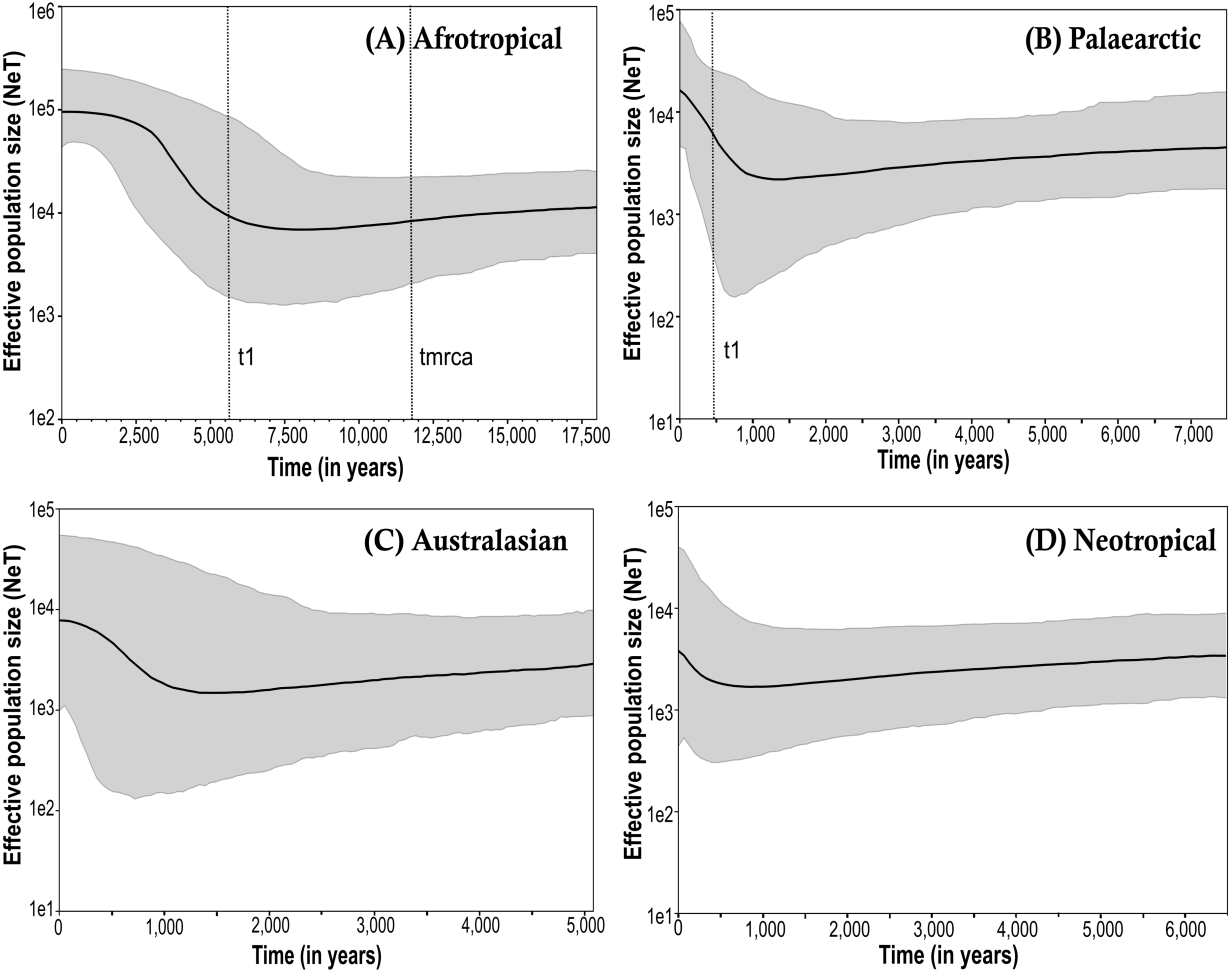
Bayesian skyline plot (BSP) estimate of Medfly demographic history for the biogeographic regions (A) Afrotropical; (B) Palaearctic; (C) Australasian and (D) Neotropical. The *X*-axis is in units of time before present (BP), and the *Y*-axis is equal to the log scale of N_*e*_T (the product of effective population size and the generation time in years). Each BSP plot described the demographic history per biogeographic region represented by a median line (solid line horizontal) with 95% High Posterior Distribution in grey (HPD; equivalent to margins of error). The dashed line t1 is the time of population expansion per biogeographic group. The Time to the most recent common ancestor (tmrca) is represented in the Afrotropical population around 11,600 BP.

The Bayesian skyline plots exhibited differences in the effective population size calculated among the biogeographical regions (Fig. 3; only the results obtained from the simulations using the corrected mutation rate of *D. melanogaster* are shown). The time to the most recent common ancestor (*tmrca*) was estimated at around 11,600 years ago in the Afrotropical (Fig. 3A). This group also showed a substantial increment (one order of magnitude) in the effective population size after the outset of the Holocene (∼10,000 years ago) suggesting a signature of recent expansion which became significant around 3,500 years ago (i.e., when the 95% highest posterior density (HPD) limits no longer includes older estimates), after which the population size was largely stable until the present time (Fig. 3A). In contrast, the Palaearctic group (Fig. 3B) had a lower effective population size and showed more recent date estimations compared to the Afrotropical group, which exhibited significant population expansion only after about 500 years ago. The Australasian and Neotropical groups (Figs. 3C and 3D) remained at a stable population size from about 1,000 years ago and then showed a slight but in significant increment.

**Figure 4:**
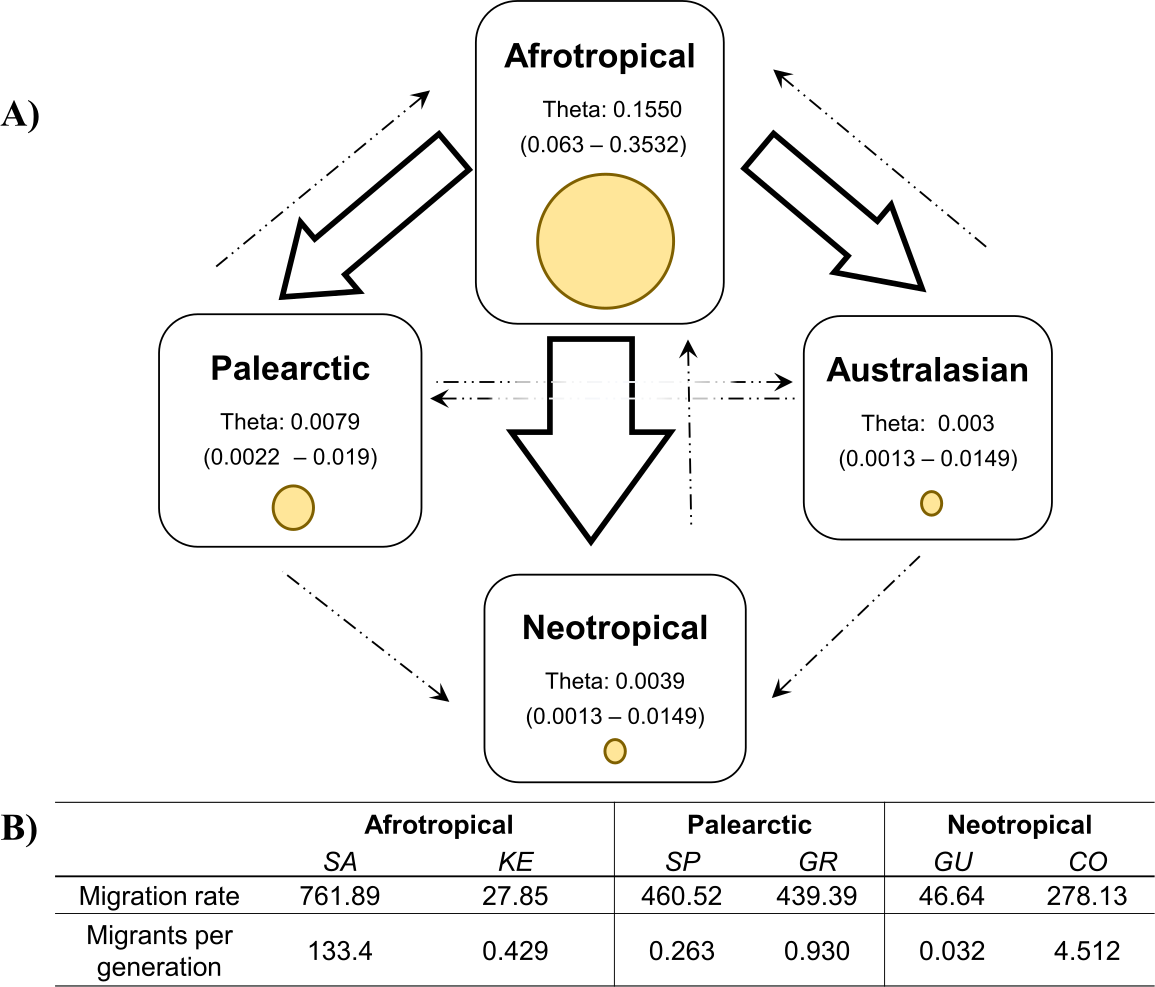
Values of theta and migration between the biogeographical regions. The figure (A) contains the theta and migration values among the four biogeographic regions. Yellow circles represent the theta value per biogeographic group and the values in brackets are the 95% HPD confidence. The arrows indicate the direction of the migration and their thickness is the proportion of the migrants per generation. (B) Migration rate and migrants per generation of specific medfly populations within the biogeographic region, country names are abbreviated as in Table 2

The coalescent analysis using Lamarc estimated a *θ* ranging from 0.1550 (Afrotropical) to 0.003 (Australasian). The Lamarc results also indicated asymmetric migration between the Afrotropical and the other populations, whereby the Palearctic received the lowest migrants per generation (Nm = 4.35) and the Australasian population the highest (Nm = 5.47), while migrant flow in the opposite direction was insignificant (Fig. 4A). Migration analyses conducted exclusively on populations from within each biogeographic region showed high levels of unidirectional exchange within the Afrotropical and Neotropical regions (Fig. 4B), such as the remarkably high Nm SA to KE =133.4 and Nm CO to GU = 4.5, while migrants were notably lower in the reverse direction (Nm KE to SA = 0.429 and Nm GU to CO = 0.032 respectively) (Fig. 4B).

## Discussion

The aim of this study was to describe the current genetic structure and recent demography of *C. capitata* and to provide insights into potential invasion routes leading to its worldwide distribution. Our extensive phylogeographic analysis has revealed a rapid colonisation process over the last 500 years, and a complex genetic structure of *C. capitata* with clear variation between biogeographic regions. The colonisation process of the medfly is well documented by both historical records (Malacrida et al., 1998; Myers et al., 2000) and molecular studies (Barr, 2009; Bonizzoni et al., 2004; Gasperi et al., 2002; Karsten et al., 2015; Karsten et al., 2013). In fact, the recreation of *C. capitata* invasion routes in this study fits broadly with that proposed in the literature, i.e., the medfly populations first migrated from the ancestral Afrotropical region to the Palearctic and then to the Australasian and Neotropical regions (Malacrida et al., 2007).

The highest genetic diversities were found in Kenya and South Africa belonging to the Afrotropical cluster. This was expected because the southeastern African countries had been identified as the medfly’s ancestral native range (De Meyer et al., 2002). Western African areas have been proposed to be part of this large native population source distributed across all of Sub-Saharan Africa (Gasparich et al., 1997), but this interpretation conflicts with our finding that the sample from Ghana shows lower genetic variation than Kenya and South Africa, and the *Fst* results are statistically significantly between Ghana and the other two populations. In the network, the Ghana haplotypes are part of the Afrotropical cluster, but all of them are unique. These findings support the existence of native, but genetically differentiated populations in West Africa, although the number of individuals (a total of five) remains too low to resolve the contradicting literature on the subject of population subdivision in Sub-Saharan Africa (De Meyer et al., 2002; Gasperi et al., 2002; Malacrida et al., 1992).

Biological invasions are often associated with a decrease in genetic diversity of the invasive species due to a small number of founder events in their introduced ranges (Lockwood, Cassey & Blackburn, 2005; Lockwood, Cassey & Blackburn, 2009). It is therefore unsurprising that we found evidence of a gradual loss of genetic variability from the ancestral Afrotropical region to the Palearctic and all other populations. Low genetic diversity was particularly obvious in the population from Israel, which was represented by the largest number of individuals of all sampled regions, and yet exhibited very low levels of genetic diversity. Similar results were reported for another mitochondrial gene ND4 (NADH subunit 4) in two different populations collected in Israel (Elfekih, Makni & Haymer, 2010). Iran and Greece also had low genetic variation compared to other populations, possibly because of limited hosts and climatic ranges suitable for the medfly in these regions. In addition, the constant eradication efforts in these countries, and in particular the use of SIT, might have resulted in occasional population bottlenecks and reduced genetic diversity in these populations. In contrast, the populations from Spain were the most genetically diverse in the Palearctic. The finding may in part be affected by the origin of the Spanish specimens, which were from multiple sites and thus may contain local variation that is not incorporated at most other country samples. In addition, populations in Spain might have a longer phylogeographic history and thus greater diversification, as the likely entry point to the Mediterranean basin.

The curious presence of a shared haplotype (Cc_21) among extremely distant populations suggests a recent connection of all non-native populations. This common haplotype is also present in South Africa, but only as a very small proportion, and it is peripheral to the haplotype network of African haplotypes. While the distribution of Cc_21 indicates a shared history of all non-native populations, the derivation of this haplotype from the South African population is not strongly supported. An individual carrying the Cc_21 haplotype could be the ancestor to all non-native populations, even if this haplotype was rare in the ancestral population, but conceivably the source population could be from elsewhere in Africa where the Cc_21 haplotype is more prevalent. Only more detailed surveys of native African populations will resolve this question.

The interpretation of demographic history results differs among the biogeographic region. The Bayesian Skyline plot result for the Afrotropical region showed it to be the most ancient population dated to about 11,600 years ago. Nevertheless, this time frame is far younger than ages usually associated with the time since species originated, or even the age of a closely similar tephritid fossil found in the Dominican Republic dated to the mid-Miocene to early Eocene (Norrbom, 1994). However, the signature of population expansion coincides with a period when the region underwent the African Humid Period which is characterised by major climatic changes that influenced ancient human settlements (Manning & Timpson, 2014). In this context, new strategies for plant use were developing in Africa about 17,000 years ago, although plant domestication was recognised only later at around 4,000 BP (Marshall & Hildebrand, 2002). In the Afrotropical region, the significant expansion signature detected about 3,500 years ago by the Bayesian skyline plot is coincident with the plant domestication period in the region. Currently, population size in this region is stable as we can see in the plot but also can be supported by the negative Tajima’s *D* and Fu’s values for Kenya and South Africa which are best interpreted as the result of purifying selection, as expected in mitochondrial protein-coding gene evolution (Meiklejohn, Montooth & Rand, 2007).

On the other hand, in Spain as one of the first points of colonisation and presumed early origin of Mediterranean populations, the patterns of COI variation may be explained by purifying selection. In contrast, the Palearctic BSP showed a population expansion, as also described before in this region (Reyes & Ochando, 2004). Non-synonymous substitutions, which are generally rare in mitochondrial genes, especially in the cytochrome oxidase genes (Pentinsaari et al., 2016), were found in all regions, but predominantly in introduced populations characterised by non-significant neutrality tests as expected for star-like topologies. Their position near the tips of the haplotype network suggests that they correspond to neutral variation or slightly deleterious mutations that are maintained in fast expanding populations, rather than adaptive changes affecting, for example, the metabolic rate due to the new environmental conditions exposed (Castoe et al., 2008), and thus these changes are consistent with the inference of fast population expansion.

The migration patterns within geographic regions might be affected differently in various parts of the world (Fig. 4). For example, in the Neotropics pest management differs notably among the countries. Guatemala is recognised for the successful establishment of the Mediterranean Fruit Fly Eradication Program (Moscamed Program) which is effectively containing the medfly within Central America (Aluja & Liedo, 1993; Enkerlin et al., 2017). A notable success of this program was the development of the Sterile Insect Technique (SIT) (Szyniszewska & Tatem, 2014). On the other side, Colombia has also recognised the presence of the medfly (ICA, 2010) and triggered the National Fruit Fly program focusing on detection, control and eradication methods of medfly based on mass trapping and chemical application (Conpes, 2008; Lasprilla, 2011). Despite of that, these phytosanitary efforts are not enough to reduce the potential pest risk of Colombian commodities (PPHIS, 2018; Szyniszewska et al., 2016) and the COI study produced clear evidence for the unidirectional migration from Colombia to Guatemala. Given the differences in their pest control management methods, there is a risk that high migration rate from Colombia now interferes with the successful Guatemalan program established some 40 years ago.

## Conclusions

The colonisation process of the medfly appears to be associated with a relatively stable demographic structure separating the Afrotropical region and the introduced range (Palearctic, Neotropical and Australasian), but characterised by residual levels of connectivity at regional scales despite considerable distance separating the populations, such as Egypt and Iran or Brazil and Peru. However, the COI marker used in this study has limitations due to comparatively low variation that may be insufficient to resolve events on the time scale of the medfly dispersal. Yet using an appropriate mutation rate, the demographic analysis produced plausible scenarios associated with the Holocene era, which is closely related to the agriculture and domestication process in the humanity. The inferred migration patterns among populations provide crucial information for the understanding of successful medfly invasions and thus pinpointing where countermeasures are required, in particular in a world connected via agricultural commodities trade. The case of successful containment in Guatemala and the dangers of fruit fly migration from elsewhere in the South and Central American regions illustrate these problems clearly. We used the most basic of molecular markers to study these phenomena, based on short fragment of a single locus, and studied pattern and process of medfly history at global levels based on just 14 local sites. The results are highly plausible and consistent with other studies using diverse approaches. However, the conclusions have to remain tentative, given the limited detail of sampling. Genomic approaches and much denser sampling at regional and global scales will be required to confirm the conclusions drawn here.

##  Supplemental Information

10.7717/peerj.5340/supp-1 Table S1 Unique haplotypes based on 51 sequences of the cytochrome oxidase gene I (COI) included in this study N, number of individuals per haplotype; Haplotype code, corresponding to each unique haplotype nomenclature.

10.7717/peerj.5340/supp-2 Supplemental Information 1 Unique haplotypes of the cytochrome oxidase gene I (COI) obtained from the sequencing analysis of 352 medfly specimens collected in 11 sites across the world included in this study Cc correspond to *Ceratitis capitata,* and the number refers to the region where the specimens belong as found in Fig. 1 (haplotype network).

## Additional Information and Declarations

### Data Availability

The following information was supplied regarding data availability: Genbank: MH628362–MH628394.
