# Supplementary material for: Population genetics and migration pathways of the Mediterranean fruit fly Ceratitis capitata inferred with coalescent methods
Source: PeerJ. 2018 Aug 7;6:e5340. doi: 10.7717/peerj.5340 (PMC6086102; doi:10.7717/peerj.5340)
Supplement: Table S1 — N, number of individuals per haplotype; Haplotype code, corresponding to each unique haplotype nomenclature. [file peerj-06-5340-s001.docx]

| **Biogeographic region** | **Samples site** | **State/Province/Locality** | **N** | **Haplotype code** | **Genbank accession number** |
| --- | --- | --- | --- | --- | --- |
| Afrotropical | Kenya | ND | 1 | Cc01 | GQ154189 |
|  |  | Ruiru | 1 | Cc02 | JN705010 |
|  |  | Ruiru | 1 | Cc03 | JN705011 |
|  |  | Ruiru | 2 | Cc04 | JN705012, AY788415 |
|  |  | Kibwesi | 1 | Cc05 | JN705013 |
|  |  | Western Highlands | 1 | Cc06 | JN705014 |
|  |  | Watamu | 1 | Cc07 | JN705015 |
|  |  | Ruiru | 1 | Cc08 | JN705016 |
|  |  | Nairobi | 1 | Cc09 | JN705017 |
|  |  | Western Highlands | 1 | Cc10 | JN705018 |
|  |  | Ololua forest | 1 | Cc11 | JN705019 |
|  |  | Watamu | 1 | Cc12 | JN705020 |
|  |  | Kakamega, Ngong Road Forest, Msambweni, Ololua forest | 4 | Cc13 | JN705022, JN705025, JN705028, JN705041 |
|  |  | Arabuko Sokoke Forest | 1 | Cc14 | JN705023 |
|  |  | Nairobi | 1 | Cc15 | JN705024 |
|  |  | Sabaki | 1 | Cc16 | JN705026 |
|  |  | Voi | 1 | Cc17 | JN705027 |
|  |  | Ololua forest | 1 | Cc18 | JN705042 |
|  | Ghana | Kade | 1 | Cc32 | JN705034 |
|  |  | Kade | 2 | Cc33 | JN705035, JN705037 |
|  |  | Kade | 1 | Cc34 | JN705036 |
|  |  | Kade | 1 | Cc35 | JN705038 |
| Palearctic | Iran | ND, Amol, Behshahr, Jouybar, Neka, Nur, Sari, Tonekabon, Qaemshahr | 10 | Cc21 | JQ668128, KM660641-KM660643, KM660646, KM660648- KM660652 |
|  |  | Neka | 1 | Cc40 | KM660645 |
|  |  | Neka | 1 | Cc41 | KM660647 |
|  | Greece | Thessaloniki, Aetolia-Acarnania | 5 | Cc21 | HQ677179-HQ677182, HQ677184, |
|  |  | Thessaloniki | 1 | Cc42 | HQ677183 |
|  | Spain | Canary Islands | 1 | Cc21 | GQ154188 |
| Neotropical | Guatemala | Antigua, Mazatenango | 4 | Cc21 | HQ677174-HQ677177 |
|  | Brazil | ND | 1 | Cc57 | DQ116363 |
